# Tissue- and Sex-Specific Molecular Signatures of Aging and the Impact of Developmental Programming

**DOI:** 10.1093/geroni/igaf122.1532

**Published:** 2025-12-31

**Authors:** Michael Olivier

**Affiliations:** Wake Forest University School of Medicine, Winston-Salem, North Carolina, United States

## Abstract

Aging, even in healthy individuals, induces a wide range of molecular changes in cells, tissues and organs, resulting in (and mediating) the age-related decline in health. Many of these changes are influenced by both genetic and lifestyle factors, including early exposures during fetal development. We have used baboons, a non-human primate species highly similar in genetics, physiology and nutrition to humans, to characterize age-correlated molecular changes in organs and tissues using in integrated multi-omics approach. We have demonstrated that different organs show early age-related changes at different timepoints, and in a sex-specific manner, highlighting differences in aging and age-related changes to molecular and cellular functions. Also, early molecular changes are tissue-specific, with different genes, proteins, metabolites and pathways affected. Examining the adult offspring born to mothers that are either under-nourished or over-nourished during pregnancy, we also have evidence that these early exposures substantially alter the aging trajectory, lead to different molecular and pathway changes on the cellular level, and result in unique characteristic age-related changes in organ function. This suggests that developmental programming (the exposure in utero) not simply accelerates aging, but fundamentally alters the molecular mechanisms mediating age-related effects in these animals.

